# Genome-wide molecular fingerprinting reveals local geographical genetic patterns in the North American angiosperm genus *Triosteum* (Caprifoliaceae)

**DOI:** 10.1371/journal.pone.0325657

**Published:** 2025-06-16

**Authors:** Tom Ruttink, Bryan Connolly, Kurt Lamour, Jonathan Hulvey, Yves Bawin, Erin C. Hilley, Peter Tandy

**Affiliations:** 1 Plant Sciences Unit, Flanders Research Institute for Agriculture, Fisheries and Food (ILVO), Melle, Belgium; 2 Department of Plant Biotechnology and Bioinformatics, Faculty of Sciences, Ghent University, Ghent, Belgium; 3 Department of Biology, Eastern Connecticut State University, Willimantic, Connecticut, United States of America; 4 Department of Entomology and Plant Pathology, University of Tennessee, Knoxville, Tennessee, United States of America; 5 Meise Botanic Garden, Meise, Belgium; 6 Natural Resources Program, Massachusetts Army National Guard, Camp Edwards, Massachusetts, United States of America; University of Florida Tropical Research and Education Center, UNITED STATES OF AMERICA

## Abstract

*Triosteum* (Caprifoliaceae) is a genus of herbaceous perennial angiosperms composed of three Asian and three North American species. The range of two similar species, *T. aurantiacum* and *T. perfoliatum*, overlap in the state of Massachusetts, USA, where the latter species is considered locally endangered and protected by regulations. The population occurring in Barnstable Co. MA, USA was morphologically atypical and had intermediate characteristics between the two taxa. Genome-wide molecular fingerprinting was used to identify this population by comparison to nearby populations with typical morphology for each of the reference species *T. aurantiacum* and *T. perfoliatum*. A set of 220,518 high quality SNPs were used to calculate expected heterozygosity, *F*_*IS*_, nucleotide diversity, and *F*_*ST*_, and to create short multi-allelic haplotype markers. Detailed population characterization was performed using Principal Component Analysis (PCA) and fastSTRUCTURE analysis on SNPs, and the haplotype markers were used to create a NJ phylogenetic tree, and pairwise comparisons of the genetic distance (Jaccard Inverse Distance) between individual plants within and between subpopulations, populations, and species. Furthermore, complete chloroplast genome sequences were created, and structural polymorphisms characterized and compared to a range of closely related species. Taken together, the data reveals a fine subpopulation structure within the morphologically atypical population at Barnstable Co. MA, that are more closely related to *T. perfoliatum* than to *T. aurantiacum.*

## Introduction

*Triosteum* (Caprifoliaceae) is a genus of herbaceous perennials composed of six species, three of Asian [1] and three of North American origin [2], though the delineation of the North American taxa has shifted over time. Linnaeus initially described two species in the region, *T. perfoliatum* L. and *T. angustifolium* L., whereas Rafinesque, in 1836, described six North American species [3]. The two-species classification was favored until E.P. Bicknell described a third species *T. aurantiacum* Bickn. in 1901 [3]. The identity and distinctiveness of *T. aurantiacum* was later called into question in 1923 by K.M. Wiegand, though the three-species classification is currently used in the modern botanical description of the region [4–6]. An additional taxon, a named hybrid or nothospecies was added in 2010; *T. angustifolium* var. *eamesii* was revealed to be a hybrid of *T. angustifolium* and *T. aurantiacum* and is currently known as *T.* × *eamesii* (Wiegand) [7].

**Fig 1:**
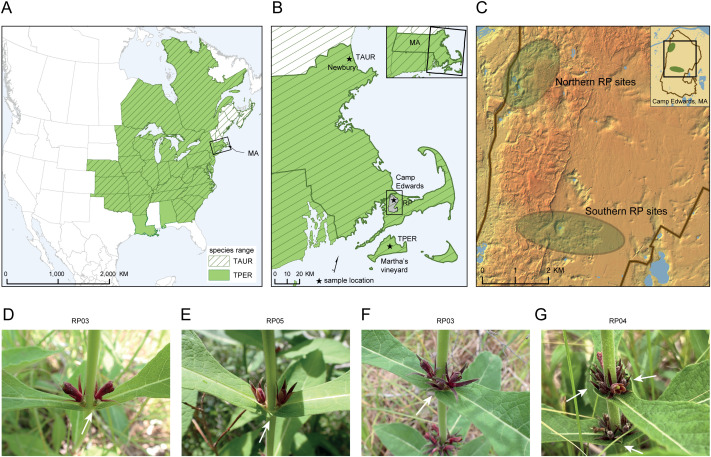
Species range distribution and leaf morphology. **A)** Species range map for *T. aurantiacum* (TAUR) and *T. perfoliatum* (TPER). **B)** Locations in Massachusetts where TAUR, TPER, and Rare Plant (RP) plant samples were collected. **C)** General locations of the two *Triosteum* sub-populations (Northern group and Southern group) at Camp Edwards where RP sites are located (i.e., RP13). The northern RP sites include RP13 and RP15 and the southern RP sites include RP03, RP04, RP05, RP06, and RP07. **D)** A plant from RP03. The leaves at a middle node are not fused around the stem, indicating TAUR leaf morphology. **E)** A plant from RP05. The leaves at a middle node are fused, although not broadly, around the stem, not clearly indicating TAUR or TPER leaf morphology. **F)** A plant from RP03. The leaves at a middle node appear more broadly fused around the stem on this plant than D or **E)**, suggesting TPER leaf morphology. **G)** A plant from RP04. The leaves at the middle nodes are definitively broadly fused around the stem, indicating TPER leaf morphology.

The three species are widespread throughout Eastern North America and inhabit mostly woodland and woodland openings but can also be found on talus slopes and in meadows or prairies [4,5]. Though the species are widespread within their ranges, and none are listed as endangered or imperiled at a national level in Canada or the United States, many states or provinces consider them rare locally. *Triosteum perfoliatum* is found in 30 US states, two Canadian provinces (Fig 1A), and imperiled in 11 of those regions; *T. angustifolium* can be found in 25 US states and one Canadian province and imperiled in 14 regions; and *T. aurantiacum* (being the most widespread) is found in 29 US states, four Canadian provinces (Fig 1A), and imperiled in eight regions [8]. Correct identification of these species has conservation, legal, and economic consequences if one species within the genus is regulated as an endangered species and the others are not. Misidentification can lead to accidental extirpation of a locally rare plant on one hand or lead to unnecessary regulation or prohibition of human activities, e.g., economic development, military training activities, or municipal development, on the other.

This study investigates the taxonomic identity of a population of *Triosteum* found at Camp Edwards, a Massachusetts Army National Guard (MAARNG) training area located on Joint Base Cape Cod (JBCC), in Barnstable Co., Massachusetts, USA (Fig 1B, C). Kettle holes or individual depressions in the landscape made as the glaciers receded that support *Triosteum* and other rare plants at Camp Edwards (i.e., *Ophioglossum pusillum*, state-listed threatened) are called Rare Plant (RP) sites (Fig 1C). The *Triosteum* population at Camp Edwards appears morphologically intermediate between *T. perfoliatum* and *T. aurantiacum*.

One of the key features that distinguishes the two species is *T. perfoliatum* having strongly perfoliate leaf bases at least at the middle nodes of the plant in comparison to *T. aurantiacum*, which has sessile leaf bases [4,5] (Fig 1D–G). The Camp Edwards plants have baffled regional botanist for decades in that they display within the population strongly perfoliate to weakly perfoliate leaf morphologies and sessile leaf bases but are otherwise morphologically similar (Fig 1D–G), therefore the population does not clearly key to one species or the other. F.C. Lane in his 1954 dissertation captured the essence of the problem: “It is not at all uncommon in examining a series of herbarium specimens to find two plants with identical collection data labeled as two different species because there is a slight variation in the shape of the leaves” [9]. *Triosteum perfoliatum* is listed as endangered under the Massachusetts Endangered Species Act and protected under state law [10]. Consequently, this means that proper taxonomic classification of the population influences land use planning and resource allocation, including monitoring and protection, on Camp Edwards. *Triosteum angustifolium* and the hybrid *T.* × *eamesii* also exist in New England, but the closest known individuals of *T. angustifolium* are found approximately 200 km to the west of the study population at Camp Edwards, and it is unclear whether natural populations represent the ‘pure’ species. In addition, there is no morphological evidence that *T. angustifolium* is closely related to the Camp Edwards plants. Lastly, if pure *T. angustifolium* populations exist in the region, they are extremely rare, and sampling may negatively affect population integrity.

The objectives of this study were two-fold. First, because a varied number of *Triosteum* species have historically been described in the region and morphological characterization provides inconclusive evidence for taxonomic classification to either *T. perfoliatum* or *T. aurantiacum*, the Camp Edwards population was genetically compared to geographically proximate reference samples of *T. perfoliatum* and *T. aurantiacum* that we were permitted to collect from. Second, we used molecular genetic fingerprinting based on whole-genome shotgun sequencing, identification of polymorphisms in the nuclear and chloroplast genome, comparison of the *within* and *between* population genetic distances and delineation of phylogenetic relationships and population substructure to study the taxonomic identity of these plants and to provide a preliminary investigation of population genetic diversity in this understudied genus in southern New England.

## Materials and methods

### Plant sampling

A *Triosteum* population of unknown taxonomic origin was sampled (n = 26 individuals) at Camp Edwards (41.654501° - 70.536865°). Plants were sampled from the northern and southern areas of the base and represented a total of seven subpopulations or RP sites (Fig 1C). RP sites were labelled with a rare plant population number (e.g., RP06), and each suffix number corresponds to individual plants found within one kettle hole depression (e.g., RP06-32). Plants with varying degrees of the perfoliate leaf trait were sampled, thus representing both perfoliate and sessile leaf types as observed at Camp Edwards. Two other reference *Triosteum* populations were sampled for genetic comparison. One population with clear *T. aurantiacum* morphology was sampled (n = 7 individuals, named TAUR) from Martin H. Burns Wildlife Management Area in Essex Co. Newbury, Massachusetts USA (Fig 1B). Additionally, cuttings of *T. perfoliatum* (n = 5 individuals, named TPER) with strongly perfoliate leaf bases that were propagated from a natural (wild) population on Martha’s Vineyard, Dukes Co. Massachusetts were provided by the Polly Hill Arboretum (Fig 1B). The RP13 and RP15 samples were collected from the northern RP sites and RP03, RP04, RP05, RP06 and RP07 samples were collected from the southern RP sites. All samples were taken in August 2020. Because *Triosteum* individuals can produce multiple stems per genet and ramets are clustered, only one leaf sample per stem cluster was taken to avoid sampling the same individual twice. Leaf samples were removed from the plants and immediately placed on ice and freeze dried within 48–72 hours.

### Genomic DNA extraction and whole genome shotgun sequencing

Whole freeze-dried leaves were ground to a fine powder using a sterilized mortar and pestle. Genomic DNA was extracted from approximately 20 mg of the tissue powder using the MagMAX DNA Multi-Sample Ultra Kit 2.0 (ThermoFisher Scientific, USA) according to the manufacturer’s instructions and the extracted DNA was visualized using an Egel 2% EX cartridge (Thermofisher Scientific, USA) and quantified using a Qubit device and the 1X dsDNA High-Sensitivity kit (ThermoFisher Scientific, USA) according to the manufacturer’s instructions. Approximately 500 ng of the resulting high molecular weight DNA was shipped overnight to Admera Health LLC (South Plainsfield, NJ) for construction of PCR-free, dual-indexed Illumina libraries according to the protocols provided by Illumina and sequenced on an Illumina HiSeqX device running the PE-150 configuration. Adapter sequences were removed from raw read sequences using Cutadapt v3.3 [11], retaining reads with minimum read length of 40 base pairs (bp). FASTQC [12] analysis showed high quality base calling in the data sets. We matched the minimum read length to the sliding frame length for haplotype calling (40 bp) using our SMAP software (see below). On average, only 0.25% of reads per sample were shorter than 40 bp after adapter trimming (See S1 Table and S1, S2, S3, and S4 Fig for further details on read quality and mapping statistics).

### Nuclear and chloroplast reference genome assembly

Because there was no reference nuclear genome sequence publicly available for *Triosteum*, and we wanted to use a reference sequence that was as closely related as possible to our samples (to facilitate optimal read mapping (BWA-MEM), reference-based SNP calling (GATK) and haplotype calling (SMAP)), a draft reference genome sequence was constructed separately for the TAUR-05, TPER-03 and RP15-03 samples using trimmed PE-read data and the DeBruynGraph-based *de novo* assembler implemented in CLC Genomics Workbench with default settings and minimal contig length of 500 bp. BLASTn analysis with the full chloroplast genome sequences of *T. himalayanum*, *T. pinnatifidum* and *T. sinuatum* (NC_045219.1, NC_037952.1, MW526077, respectively; [1]) were used to identify contigs corresponding to the chloroplast genome sequence for TAUR-05, TPER-03 and RP15-03. The respective chloroplast contigs were separated from the nuclear *de novo* assembly contigs, and were concatenated in the correct order according to the BLASTn hits, taking the internal duplicated region structure into account [1] to initially create three draft chloroplast reference genomes for *T. perfoliatum*, *T. aurantiacum* and individuals from the RP sites. Junctions between contigs were iteratively and manually adjusted based on detailed visual inspection of WGS read mappings per species until reads showed perfect mapping support across the entire length of the chloroplast genome assembly. Finally, whole chloroplast genome sequence alignments were created with mVISTA and a corresponding NJ phylogenetic tree was created with CLCbio Genomics Workbench, showing overall high chloroplast genome sequence conservation across previously published *Triosteum* species [1] and the three novel assemblies. In total, 11 SNPs, five 1-bp Indels, and three regions (51 bp, 51 bp, and 192 bp) that were specifically duplicated in a particular species were identified between TAUR-05, TPER-03 and RP15-03 chloroplast assemblies. These polymorphisms were manually scored for all 38 samples by mapping a random set of 10 M PE-reads per sample onto the central TAUR-05 chloroplast reference genome combined with all nuclear genome contigs, and visual inspection of the 19 polymorphic chloroplast loci in the CLCbio Genomics Workbench genome browser. Chloroplast gene models for genes (CDS), tRNA, and rRNA encoded in *T. sinuatum* (MW526077) were transferred via coordinates of the whole chloroplast genome alignments, manually curated, and these final high-quality reference sequences were submitted to genbank (*T. aurantiacum*: PQ559726; *T. perfoliatum*: PQ559726; RP15-03: PQ559728).

### Genotype calling

Adapter trimmed reads of all individual samples of the study were mapped against the TAUR-05 reference genome assembly using the BWA-MEM algorithm with all default settings in BWA v0.7.17 [13]. Next, we used various components of the SMAP package as applied to haplotype calling in WGS data [14]. The SMAP package source code is available on GitLab (https://gitlab.ilvo.be/genomics/smap-package/smap) and a detailed description of the working procedure and guidelines are available in the online User Manual (https://ngs-smap.readthedocs.io/en/latest/home.html). Sliding frames of 40 bp were created with the SMAP utility tool *sliding-frames* (frame size 40 bp, between-frame distance 40 bp). Next, regions with sufficient read depth (minimal 8 reads completely spanning the sliding frame) per locus per sample across the set of 38 samples (i.e., locus completeness of >90%, to ensure balanced representation of genetic diversity across all samples) were searched with SMAP *haplotype-sites*, yielding a total of 291,190 loci, which together represented approximately 2.7% of the total genome sequence assembly length (425.7 Mbp). SNPs were identified on those loci using the GATK 4.2.0.0 HaplotypeCaller [15] with default settings for diploid organisms. The resulting SNP data was processed according to GATK best practices using VariantFiltration and SelectVariants with the following parameters: quality by depth < 2.0 (estimated for multiple samples), Fisher strand value > 60.0, strand odds ratio > 3.0, mapping quality < 40.0, mapping quality rank sum < −8.0 (according to the GATK best practices workflow; [16]. We did not filter SNPs based on LD, nor on minor allele frequency, because we wanted to keep the possibility that individual samples showed unique SNPs or haplotypes. A total of 537,933 polymorphic, bi-allelic SNPs were retained after these filter steps. WAXY exon 10−11 fragments of *T. sinuatum* (AF277644.1), *T. angustifolium* (AF277643.1), *T. aurantiacum* (AF277641.1), *T. perfoliatum* (AF277642.1), *T. himalayanum* (AF277637.1), and *T. pinnatifidum* (AF277634.1), were retrieved from NCBI and BLASTn was performed to identify the corresponding regions in *de novo* assemblies per sample. A single SNP polymorphism was identified within the sample set, and genotype calling was based on read mapping of all reads per sample onto the TAUR-05 reference genome at the WAXY locus at contig_31377 SNP position 34123.

### Haplotype calling

Selected SNPs obtained by GATK were used for read-backed haplotype calling using SMAP *haplotype-sites*. A set of 38 indexed BAM files with mapped reads, a custom BED file with sliding frame start and end point positions (291,190 loci, see above), and a VCF file with 537,933 selected SNP positions then served as input for the module SMAP *haplotype-sites*. SMAP *haplotype-sites* evaluates the read-reference alignment at each polymorphic position within a locus and creates a short haplotype string per read that combines the calls of neighboring polymorphisms (SNPs) across the genome region covered by the sliding frame. It then counts the read depth per unique haplotype per locus, quantifies the relative haplotype frequency per locus, transforms that to discrete dosage calls using frequency interval boundaries and finally outputs the discrete haplotype dosage call table. Thus, SMAP *haplotype-sites* was used to extract multi-allelic haplotypes using read-backed phasing of the reference-aligned reads, using the following parameter settings for discrete haplotype dosage calling in diploid individuals: --mapping_orientation ignore --plot_all -u ““ --no_indel --partial exclude --mapping_quality 30 --min_read_count 8 -f 10 --discrete_calls dosage --frequency_interval_bounds 15 15 85 85 -z 2. After SMAP *haplotype-sites*, only loci (n = 114,404) with at least 8 reads per locus per sample (in total across all haplotype variants) in >90% of the 38 samples were retained to ensure genotype call completeness across the haplotype table and loci with >90% correctness (discrete dosage of 2 alleles for diploid organisms) across the sample set were retained to ensure high quality genotype calls. Thus, haplotype calling with the SMAP software provides an additional filtering procedure for high quality loci that is not possible with SNP data alone. In our haplotype analysis (which is based on physical linkage as it uses read-backed phasing within sliding frames of 40 bp), SNP markers that are linked at very short distance, end up in a single phased haplotype allele – without relying on statistical coupling (co-segregation, i.e., Linkage Disequilibrium) across samples. This reduces some of the redundancy in genetic information captured by individual neighboring SNPs that are in physical coupling phase in short haplotypes. At the same time, it increases genetic resolution because it can create multi-allelic haplotype markers per locus that are not necessarily distinguished using individual bi-allelic SNPs. Further information on the increased accuracy for the estimation of population genetic parameters using multi-allelic haplotype markers as compared to SNP markers of the same loci, are provided in Bråtelund *et al.*, [17]. Finally, only the 220,518 SNPs that overlap with the 114,404 high quality loci identified by SMAP haplotype-sites were retained for further analysis. Of these, 23,489 SNPs were singleton SNPs (a single heterozygous call on an otherwise fixed SNP in all samples with data). See S1 Table and S1, S2, S3, and S4 Figs for further details on read quality and mapping statistics of all subsequent steps of the procedure (trimming, mapping, selection of high-quality regions, and for genotype call missingness per sample after SNP filtering).

### Analysis of genetic diversity and genetic differentiation

In this study, we used SNPs and their derived haplotype markers in parallel because some computational methods are only compatible with bi-allelic SNPs, while others can use multi-allelic haplotype markers.

#### SNP sets.

Principal Component Analysis (PCA) was performed on the SNP calls generated by GATK using the *vcfR* (v1.12.0), *adegenet* (v 2.1.4) and *ade* (v ade4) packages in Rstudio (v4.2.1) [18]. In total, 220,518 SNPs that overlap with the 114,404 high quality haplotype-called loci identified by SMAP *haplotype-sites* were used for the PCA. A Bayesian genetic clustering analysis was conducted on the SNP data using fastSTRUCTURE [19]. The VCF file was converted into the fastSTRUCTURE input file with PGDspider v2.1.1.5 [20]. The number of subpopulations (K) was set between 2 and 9. The most optimal value of k was determined with the choosek.py script included in the fastSTRUCTURE package. Both the model complexity with the maximum marginal likelihood and the number of model components to explain structure in the data was most optimal for K = 4. The resulting Distruct plots were created with the distruct2.3.py script from the fastSTRUCTURE software. PCA and fastSTRUCTURE were also performed on the set of 197,032 SNPs after removing singleton SNPs. Expected heterozygosity (*H*_*e*_) and the inbreeding coefficient *F*_*IS*_ were calculated using the basic.stats function from the *hierfstat* package. Nucleotide diversity π was estimated per population with vcftools v0.1.17 [21] as the average of the nucleotide diversity values of each 50 kb window using the --window-pi parameter. Pairwise *F*_*ST*_ values as defined by [22] were calculated between all populations using the *pairwise.neifst* function in the R package *hierfstat* [23].

#### Haplotype sets.

The SMAP *relatedness pairwise* module with default parameters was used to calculate Jaccard’s Inversed Distance (JID) values for each pairwise sample comparison, based on the SMAP *haplotype-sites* discrete haplotype dosage call table. A Neighbor-Joining (NJ) phylogenetic tree was reconstructed based on the Jaccard Inversed Distance matrix (1 – J) with the nj function from the package APE v5.8 [24] in R (v4.3.1) using the haplotype call matrix. 200 bootstrap replicates of the JID matrix were created with the SMAP *relatedness pairwise* module and were used as additional input in the phylogenetic analysis to obtain node support values.

## Results and discussion

Camp Edwards is a relatively large forested and unfragmented expanse consisting of nearly 15,000 acres and sits at the base of Cape Cod (Fig 1A–C), a peninsula composed of moraine and outwash deposits from the Wisconsin glaciation [25]. Variable topography and sediments left behind from the glacial moraine deposits comprises the western and northern perimeter of Camp Edwards with a delta of meltwater transported sediments fanning out to the south and southeast [25]. This surficial geology is a major driver of the plant diversity at this location and Camp Edwards has many dry “kettle holes” which are bowl-shaped depressions formed by disconnected blocks of glacial ice left behind as the main ice sheet retreated. Kettle holes function as “frost bottoms” when cool dense air pools. This can cause more frequent and later season frost conditions, which further drives the unique plant diversity in these kettle holes [26,27]. The *Triosteum* populations at Camp Edwards can be found at the bottom of dry kettle holes which are geographically separated by roughly six to seven km of variable topography into two nested groups, the Southern group and Northern group (Fig 1C). The most prevalent plant communities across Camp Edwards, pitch pine – oak woodland, pitch pine – scrub oak, and scrub oak shrubland [28], tolerate the sandy nutrient-poor soils left from the retreating glaciers. *Pinus rigida* and *Quercus coccinea* and *Q. alba* are the defining overstorey tree species of Camp Edwards with *Quercus ilicifolia*, *Gaylussacia* spp., and *Vaccinium* spp., the most common understory shrubs. While the vegetation growing on the kettle hole slopes and upper surrounding vegetation is typical of pitch pine – oak forest/woodlands and related plant communities, the kettle holes host a unique association of plants that do not commonly occur elsewhere at Camp Edwards, or at least in the surrounding wooded areas upgradient of the frost bottoms, including *T. perfoliatum*, *Ophioglossum pusillum*, *Thalictrum revolutum*, *Stachys hyssopifolia*, *Salix occidentalis*, *Lycopus americanus*, and *Pycnanthemum tenuifolium* (personal observations during annual rare plant surveys conducted by MA Army National Guard, Natural Resources Program staff).

First, we investigated the taxonomic origin of a set of *Triosteum* individuals discovered at Camp Edwards by sequencing taxonomic barcoding markers. Genetic studies of endangered plant entities in other families have been done in the region to clarify taxonomic position to help inform regulators for endangered species listing status [29,30]. Analyses of the WAXY exon10 - exon11 fragments showed that all RP plants were identical to all *T. aurantiacum* plants, except RP15-01 and RP15-05 that shared a one SNP difference (contig_31377, position 34123, C/T) with all *T. perfoliatum* plants. None of these WAXY sequences corresponded to the previously published WAXY sequences for six *Triosteum* species [2].

Next, we compared genome-wide molecular fingerprints of 26 Camp Edwards *Triosteum* sp. individuals to five *T. perfoliatum* and seven *T. aurantiacum* individuals. Whole-genome shotgun (WGS) sequencing yielded 27M to 266M PE-150 reads per sample, corresponding to about 10x to 100x genome coverage at an estimated genome size of 779 Mb [31]. Read data of TAUR-05, TPER-03, and RP15-03, were used for *de novo* assembly of draft nuclear genome sequences, while chloroplast genome sequences were created and manually curated. The TAUR-05 sample had the most WGS data per sample (266M PE-150 reads), a low degree of heterozygosity, and yielded the best reference genome assembly in terms of total assembly length (425.7 Mbp), contiguity (N50 = 7892 bp; N90 = 1337 bp), minimal number of contigs (117,438 contigs with minimum contig size of 500 bp), and was therefore used as central reference for read mapping of all samples. Since not all samples were sequenced to equal depth, we first identified 291,190 loci (sliding frames of 40 bp) with minimum 8 reads per locus per sample in at least 35 out of 38 samples (>90%; near-completeness). Next, SNP identification and hard filtering with GATK yielded a total of 537,933 polymorphic SNP sites. Then, SMAP *haplotype-sites* was used to create discrete haplotype calls (i.e., ShortHaps; haplotypes created by joining neighboring SNP calls in sliding frames of 40 bp using read-backed phasing). SMAP *haplotype-sites* yielded a total of 266,257 haplotypes across 114,404 loci, with an average of 2.33 haplotypes per locus (range 1–8) across the 38 analyzed individuals. Only the 220,518 SNPs that overlapped with the 114,404 high quality haplotype-called loci identified by SMAP *haplotype-sites* (>90% completeness, > 90% correctness) were retained for further analysis. This step further filters out SNPs that were putatively misidentified by GATK, and provides an additional selection for high quality SNPs.

**Fig 2:**
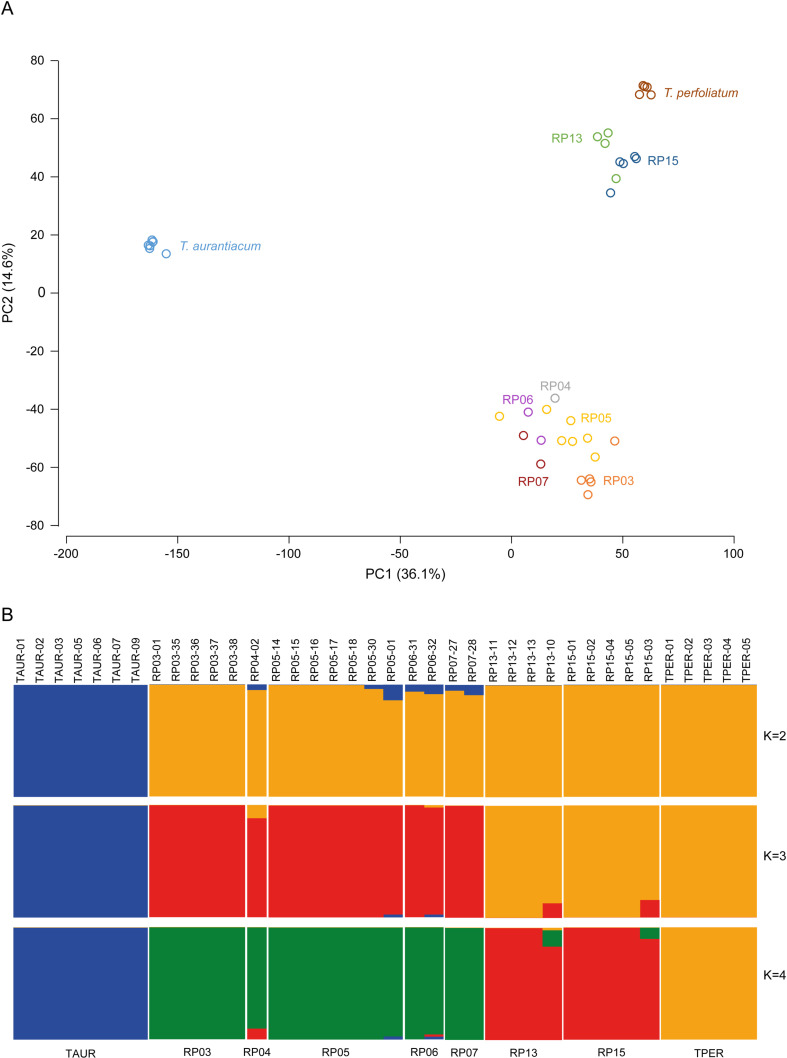
Genetic relatedness across 26 RP samples and 5 *T. perfoliatum* and 7 *T. aurantiacum* reference samples based on 114,404 loci with 220,518 high quality SNPs. **A)** Principle Component Analysis of genetic diversity. **B)** fastSTRUCTURE analysis using K = 2, K = 3, and K = 4 settings. **C)** Expected heterozygosity, inbreeding coefficient (F_IS_) and nucleotide diversity of *T. perfoliatum*, *T. aurantiacum* and RP sites. **D)** Genetic differentiation (*F*_*ST*_) between *T. perfoliatum, T. aurantiacum* and RP sites.

**Fig 3:**
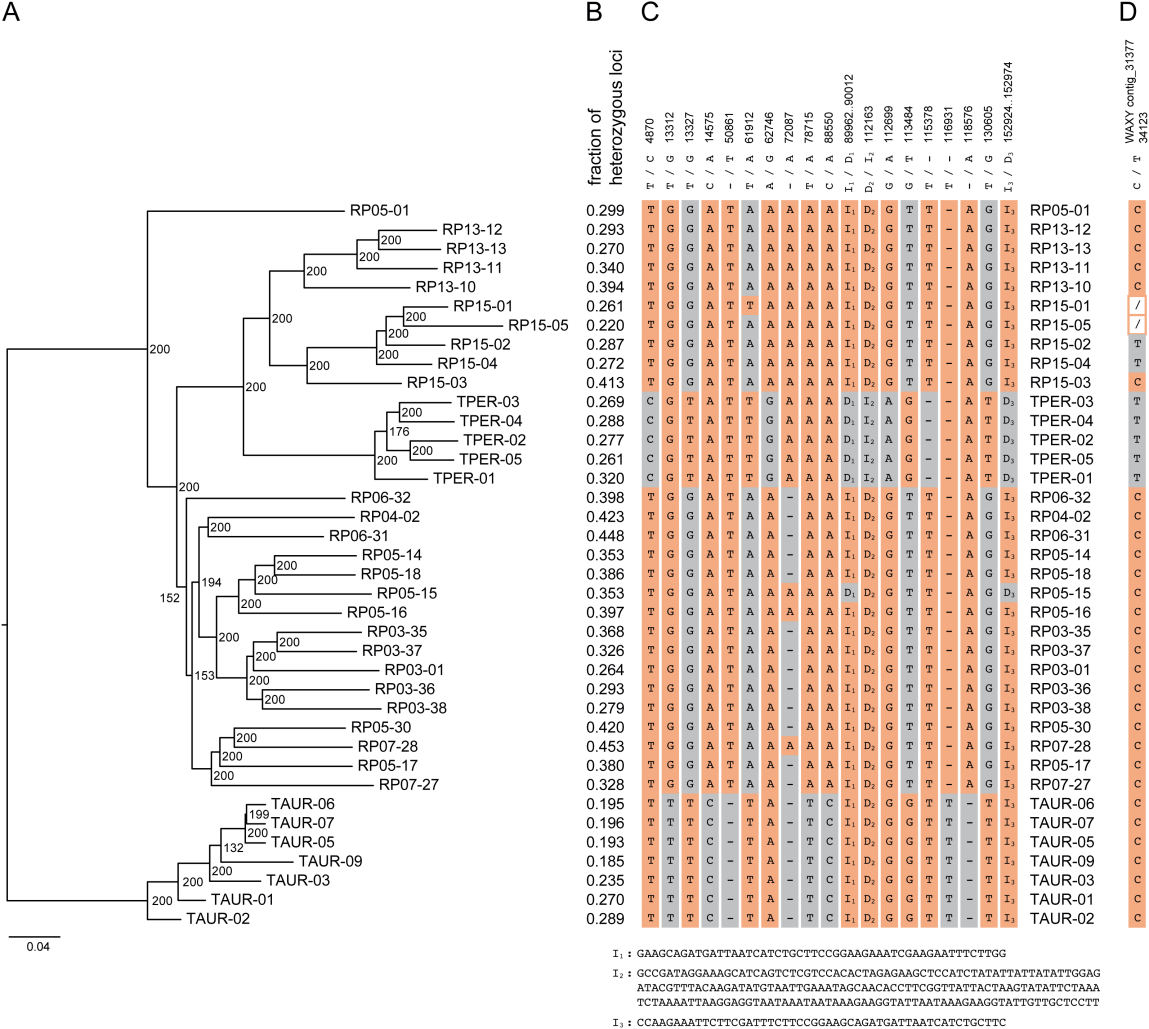
Genetic relatedness across 26 RP samples and 5 *T. perfoliatum* and 7 *T. aurantiacum* reference samples using 114,404 loci with 266,257 multi-allelic haplotypes. **A)** NJ tree based on Jaccard genetic similarities. 200 bootstrap replicates were used. **B)** fraction of heterozygous loci across all loci with data per sample. **C)** overview of structural variants (SNPs, single nucleotide Indel, and large InDels (I_1_-I_3_) across the chloroplast genome sequence per individual. **D)** variants at a single SNP in the *Waxy* locus (genotype call at the C/T polymorphism at position 34123 of contig_31377;/: missing data).

**Fig 4:**
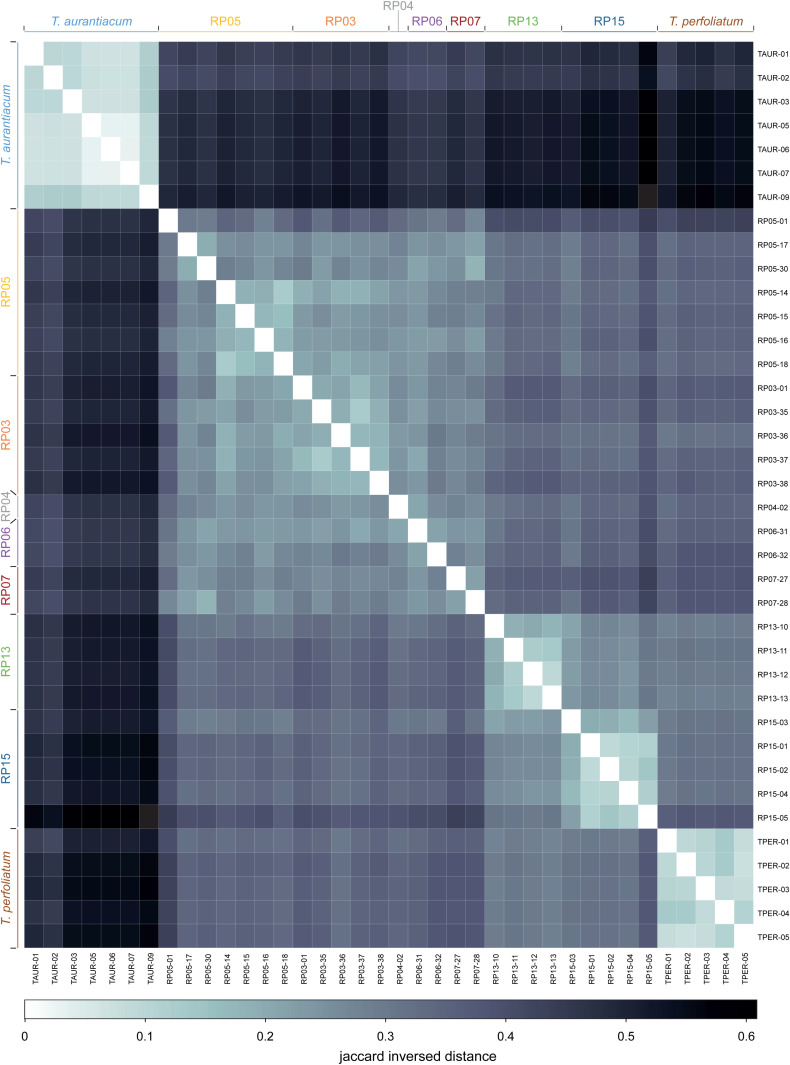
Pairwise comparisons of the genetic distance (Jaccard Inverse Distance) between individual plants within and between subpopulations, populations, and species. JID values are shown as false-color scale.

The whole-genome sequencing followed by SNP and haplotype calling methods revealed genetic differences and subpopulation structure *between* and *within* the *T. aurantiacum* (Newbury), *T. perfoliatum* (Martha’s Vineyard) and the Rare Plant site (Camp Edwards) populations. Genetic analyses were performed in parallel for SNPs (for methods that can only use bi-allelic marker data), and for haplotypes (for methods that can use multi-allelic markers). The 220,518 SNP genotype calls at the 114,404 high quality loci were used for PCA analysis (Fig 2A) to reveal global genetic similarity between and within populations, and for fastSTRUCTURE analysis (Fig 2B) to reveal subpopulation structure, and to calculate population genetic parameters such as average nucleotide diversity (π), expected heterozygosity (*H*_*e*_), and inbreeding coefficient (*F*_*IS*_) per population (Fig 2C), and the genetic differentiation between subpopulations was estimated with *F*_*ST*_ (Fig 2D). In parallel, 266,257 haplotypes across the same 114,404 loci were used to calculate a NJ tree to reveal phylogenetic relationships between samples (Fig 3A), the fraction of heterozygous loci across all loci with data per sample (Fig 3B), and pairwise Jaccard’s Inversed Distances (JID; Fig 4) to reveal genetic similarity. Taken together, all analysis consistently indicated the genetic differentiation between the *T. aurantiacum* population and the *T. perfoliatum* population, while the Camp Edwards RP populations were more closely related to *T. perfoliatum*. Within the RP sites, the RP populations from the northern sites (RP13, RP15) are more closely related to *T. perfoliatum* reference samples, while the RP populations from the southern sites (RP03, RP04, RP05, RP06, RP07) are slightly more distantly related, yet still more closely related to *T. perfoliatum* than to *T. aurantiacum*. For instance, the projected genetic variation on the first principal component (PC) axis (36.1% of genetic variation within the SNP set explained) is mostly reflected by separate grouping of the *T. aurantiacum* samples on the negative PC1 axis and the *T. perfoliatum* and RP samples on the positive axis, indicating that the reference samples can be discriminated and that the RP plants are (relatively) more closely related to *T. perfoliatum*. The second PC axis (14.6% genetic variation explained) further differentiates between a cluster of southern sites (RP03, RP04, RP05, RP06, RP07), and a cluster of *T. perfoliatum* and the two northern sites (RP13, RP15). FastSTRUCTURE analysis further confirmed the gradual differentiation. To test if filtering out low MAF (singleton) SNPs would affect the population substructure, PCA and fastSTRUCTURE were also performed with 197.029 non-singleton SNPs, yielding essentially the same results (see S5 and S6 Figs). With only K = 2 groups, all RP individuals were grouped together with *T. perfoliatum*, and separated from *T. aurantiacum*. At K = 3 groups, the northern RP individuals were still grouped together with *T. perfoliatum*, and southern RP sites started to differentiate, only at K = 4 the northern RP individuals were grouped separately from *T. perfoliatum* and southern RP sites. This was further consistent with the phylogenetic relationships revealed by the NJ phylogenetic tree as the *T. perfoliatum* clade is nested in a larger cluster with RP13 and RP15 sites, while all southern RP individuals (except individual RP05-01) are clustered in a sister clade, again clearly separated from the clade with all *T. aurantiacum* individuals. Calculation of genetic similarities as estimated using Jaccard Inversed Distances (JID) at the level of shared haplotype alleles across 114,404 loci (Fig 4, for detailed information on exact JID scores per pairwise comparison, see S7 Fig), further showed that the populations with morphology typical of *T. perfoliatum* (Martha’s Vineyard) and *T. aurantiacum* (Newbury) were genetically uniform within their population (*T. perfoliatum*: JID range 0.07–0.13; *T. aurantiacum*: JID range 0.03–0.12), and the least similar between the species (JID range 0.42–0.57), the highest values seen in this study. The Camp Edwards population is more closely related to *T. perfoliatum* plants from Martha’s Vineyard (average JID 0.34, range 0.27–0.43), than to *T. aurantiacum* plants from Newbury (average JID 0.49, range 0.40–0.61). The *Triosteum* plants from Camp Edwards show clear similarity within each RP site (RP05: average JID 0.24, range 0.12–0.35; RP03: average JID 0.18, range 0.12–0.22; RP13: average JID 0.15, range 0.09–0.20; RP15: average JID 0.15, range 0.09–0.22), and within-Northern group (RP13, RP15, average JID 0.21, range 0.09–0.31) and within-Southern group (RP03, RP04, RP05, RP06, RP07, average JID 0.25, range 0.12–0.38) subpopulations. In addition, the Camp Edwards population displays differentiation between Northern group versus Southern group subpopulations (average JID 0.35, range 0.29–0.44), two groups that were only about six to seven km apart. The genetic differentiation patterns inferred with *F*_*ST*_ (Fig 2D) confirmed the closer genetic relationship between the RP sites and *T. perfoliatum* and the separate genetic clustering of the northern and southern RP sites. The northern RP sites were not more closely related to the *T. perfoliatum* population than the southern RP sites based on the *F*_*ST*_ values, but these values might be influenced by the lower number of individuals per population included in this study. Finally, population genetic parameters revealed low levels of average nucleotide diversity in *T. aurantiacum* (0.0344*10^−3^) and *T. perfoliatum* (0.0491*10^−3^) and slightly elevated in RP sites (0.0484–0.11073*10^−3^) (Fig 2C), while negative *F*_*IS*_ values (−0.275 to −0.501) indicated relatively high levels of outbreeding across all populations, supported by expected heterozygosity of 0.125 in *T. aurantiacum* and 0.176 in *T. perfoliatum*, and in the range 0.206–0.280 within the different RP sites (Fig 2C), and the fraction of heterozygous loci across all loci with data per sample, based on haplotype calling (Fig 3B).

**Fig 5:**
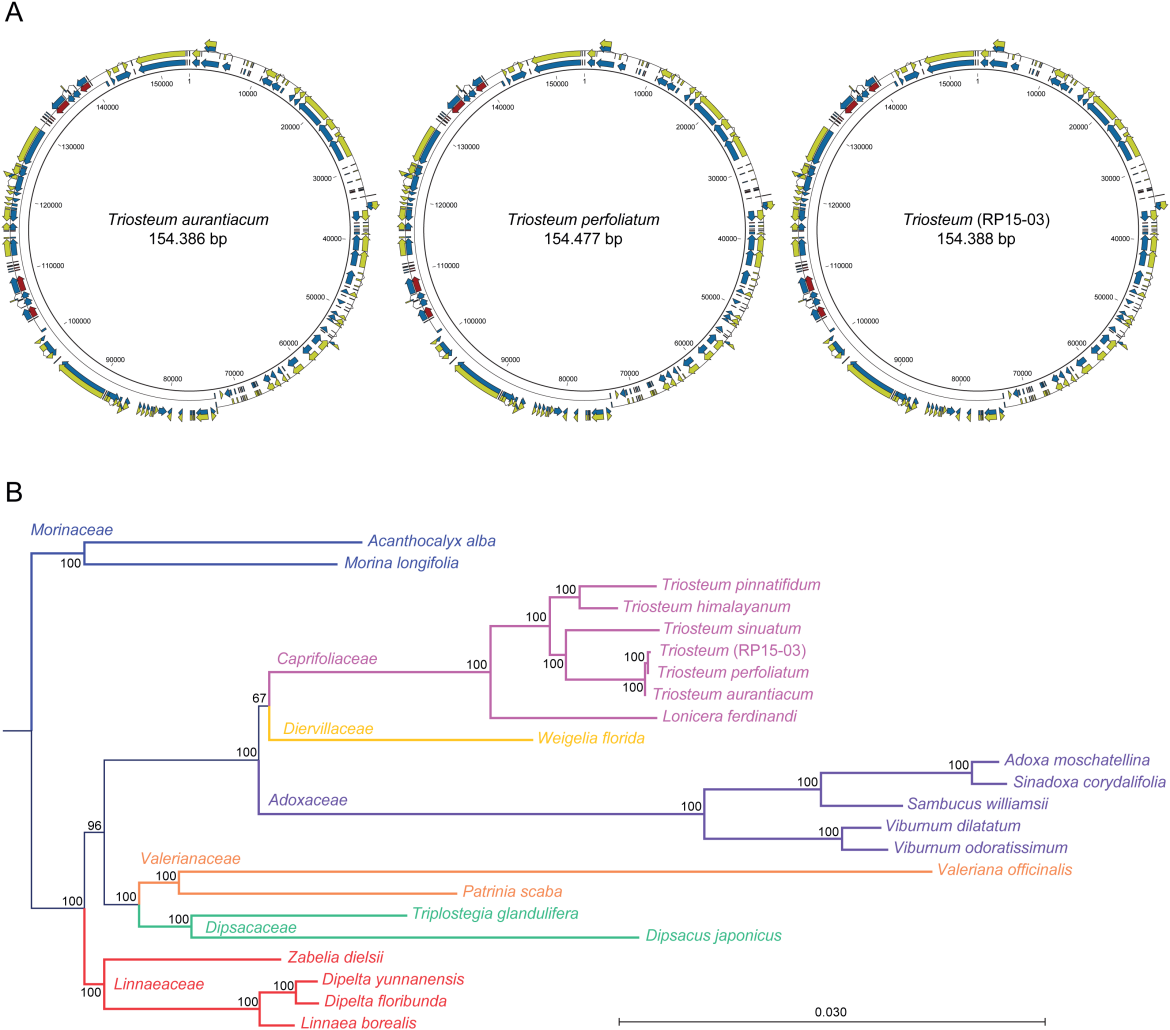
Chloroplast whole-genome assemblies of *T. perfoliatum*, *T. aurantiacum*, and RP15-03, compared to closely related species. **A)** chloroplast whole-genome assemblies with structural annotation. **B)** NJ phylogenetic tree of *Triosteum* chloroplast sequences and closely related species (accessions retrieved from Liu *et al*., 2022, whole genome alignments were created with mVISTA).

Finally, we reconstructed the entire chloroplast genome sequence of *T. perfoliatum*, *T. aurantiacum* and the RP15-03 individual (Fig 5A) and identified polymorphisms at the SNP, single-nucleotide indel and duplicated region level, for each individual (Fig 3C). This analysis showed that a total of 19 polymorphic sites exists across all 38 *Triosteum* samples studied. None of the polymorphisms was unique to one single genotype. In general, the chloroplast genome sequences were strictly conserved within the *T. perfoliatum* samples, and within the *T. aurantiacum* samples, with 15 out of 19 polymorphic sites consistently differentiating between those two populations, and the RP samples were consistently different from all *T. perfoliatum* and *T. aurantiacum* samples at the remaining four SNP loci. Conversely, the RP chloroplast consensus sequence diverged from all *T. perfoliatum* samples at 11 out of 19 polymorphic sites, and all RP samples consistently diverged from all *T. aurantiacum* samples at another set of 11 out of 19 polymorphic sites across the chloroplast genome. Furthermore, the chloroplast genome sequences of the individuals from all RP sites were also highly, but not absolutely, conserved. A single indel (72087 -/A) compared to the RP chloroplast genome consensus sequence was observed in RP05-16 and RP07-28 of the southern RP sites in which their A allele is shared with *T. perfoliatum* and the northern RP sites. Conversely, a single SNP (61192 A/T) was observed in the northern individual RP15-01 that was shared with all *T. perfoliatum* and *T. aurantiacum* samples, but no other RP individual. Finally, individual RP05-15 lacked one copy each at the two duplicated regions (D_1_ 89962–90012 and D_3_ 152924–152974; 51 bp deletions) that are typical for *T. perfoliatum*, and also shared the 72087 -/A deletion allele found in *T. perfoliatum* and the northern RP sites (Fig 3C). Taken together, these analyses showed that the RP population chloroplast genome is as divergent from that of the *T. perfoliatum* population as it is from the *T. aurantiacum* population, and that some chloroplast genome sequence diversity exists within the RP populations that is shared with either *T. aurantiacum* or *T. perfoliatum* populations. The newly assembled full-length chloroplast genome sequences were used to create a NJ phylogenetic tree (Fig 5B) with previously published chloroplast whole genome sequences and revealed the relationships to Asian *Triosteum* species and several other closely related species [1].

## Conclusion

In order to resolve the taxonomic relationship of *Triosteum* plants sampled at the Rare Plant sites at Cape Cod, we applied genetic methods with increasing levels of complexity (Fig 3): the *Waxy* locus (a single SNP within our sample set); a chloroplast whole genome sequence comparison (including SNPs, 1 bp indels and large Indels derived from duplicated regions), and nuclear genome-wide sets of hundreds of thousands of SNPs and their derived short haplotypes. These various methods did not agree on a clear and consistent grouping of the RP plants with either *T. aurantiacum* or *T. perfoliatum*. Our *Waxy* sequencing results did not agree with published *Triosteum Waxy* alleles that were previously used to distinguish between *T. aurantiacum* and *T. perfoliatum*. In our study, most RP plants were identical to *T. aurantiacum*, and only some members of the RP15 population were identical to *T. perfoliatum* at the *Waxy* locus (Fig 3D). The chloroplast genome data revealed that North American *Triosteum* is quite distinct from Asian *Triosteum*, and among high levels of chloroplast sequence conservation across all *T. aurantiacum*, *T. perfoliatum* and RP plants in general, the RP plants are as dissimilar to *T. perfoliatum* as to *T. aurantiacum*, and also showed four SNPs that were unique to the RP plants (Fig 3C). As expected, the highest resolution for population genetic analyses was achieved with genome-wide nuclear markers (Fig 3A). All population genetic measures and population structure analyses based on the nuclear genome markers all point in the same direction. The nuclear genome SNPs and haplotypes showed that the RP plants were most similar to *T. perfoliatum*, with the northern sites (RP13, RP15) more closely related to *T. perfoliatum*, and the southern sites slightly more divergent (RP03, RP04, RP05, RP06, RP07). A comparison between PCA on all 220,518 SNPs, or on 197,032 non-singleton SNPs indicated that filtering out SNPs with low MAF made no substantial difference in the interpretation of inferred patterns in populations structure in our sample set. Several observations show that populations RP04, RP05, RP03, RP07 and RP06, are more genetically diverse compared to RP13 and RP15, and display a different genetic distance to TPER and TAUR. First, individuals of locations RP04, RP05, RP03, RP07 and RP06 display higher levels of heterozygosity per sample, compared to TAUR, TPER and RP13 and RP15. Second, they display a higher Jaccard inversed distance to TPER, compared to RP13 and RP15. The phylogenetic NJ tree also shows the close clustering of TPER, RP13, and RP15, and separation of RP04, RP05, RP03, RP07 and RP06 to sister clades. However, the entire clade encompassing all RP and TPER individuals is separated from the TAUR clade. Analysis at the level of haplotype identity shared between pairs of samples shows that RP04, RP06 and RP07 share haplotypes with TAUR samples at a slightly higher percentage of loci throughout the genome, compared to the other RP individuals and TPER. However, an F1 hybridization event is expected to share at least one of its alleles for almost all loci with both progenitor species. This is clearly not the case in any of the RP samples, and the observed degree of allele sharing in RP individuals may represent weak signs of admixture of TAUR alleles into a predominantly TPER genetic background. The number of samples at RP locations and of the TAUR and TPER reference species is currently insufficient to draw conclusions on the existence of an interspecific hybridization zone, and extended sampling is required to demonstrate such fine-scale population structure. The analyses also revealed that a gradient of genetic diversity exists, both within the RP plant collection and to the respective *T. aurantiacum* and *T. perfoliatum* populations. The structured and distinct genetic identity of each subpopulation from Camp Edwards is remarkable considering the relatively small geographic scale, and the separate clustering of the Northern group versus the Southern group suggest that this species reproduces with geographically proximate closely related neighboring plants. Nevertheless, the average JID between all plants within the Camp Edwards population was 0.29, meaning they are only slightly more similar to each other than they are to the Martha’s Vineyard *T. perfoliatum* reference population (average JID 0.34), consistent with the clustering of the *T. perfoliatum* samples in a clade that was nested within all RP plants in the NJ phylogenetic tree based on nuclear haplotype diversity data (Fig 3A). Therefore, we conclude that plants from Camp Edwards are more closely related to *T. perfoliatum* than to *T. aurantiacum*.

One hypothesis to explain the high genetic differentiation between the northern and southern RP sites is that the glacial depressions or frost bottoms (here referred to as RP sites) exist as isolated habitat islands, separated from each other by unsuitable habitat conditions [32], and maximum pollinator flight distance. *Triosteum* flowers attract long-tongued pollinators, especially bumblebee (*Bombus* spp.) and Anthophorid bees (*Anthophora* spp.) (USDA-USFS, 2025). According to a study conducted by [33] using micro radio telemetry, the maximum flight distances of three European bumblebee species (*Bombus* spp.) were found to be 2.5 km, 1.9 km, and 1.3 km, respectively. Six *Bombus* species and one rare *Anthophora* species have been documented at Camp Edwards that could be potential pollen vectors. The northern and southern RP site complexes are separated by roughly 5–7 km. If the maximum flight distances of *Bombus* in North America are comparable to those observed in Europe, the distance between the northern and southern sites exceeds the typical range for a bumblebee by two to three times. The distance, isolated depressional features, and the circumstance that many *Triosteum* locations are over shaded by a dense cover of bracken fern (*Pteridium aquilinum*) by the time of flowering may create conditions difficult for pollinators to visually locate the plants. Seed dispersal by animals may also have its limitations between northern and southern RP sites since odds are low that the seeds of ingested fruit at a northern RP site would be subsequently deposited 5–7 km away at southern RP sites, and *vice versa*. Dispersal limitations of pollen and seeds by animals to spatially isolated sites that provide the specific conditions necessary for recruitment may explain genetic differentiation between northern and southern RP sites.

In the future, more populations of *T. aurantiacum* and *T. perfoliatum* could be added to investigate if admixture has occurred between the species, creating a species-continuum. These findings suggest that it is difficult to define thresholds that strictly separate species based on genetic similarity or population structure analysis, and prompt further research as the current number of reference samples for *T. aurantiacum* and *T. perfoliatum* now appear too small to represent the range of genetic diversity within the larger geographic area surrounding Camp Edwards. A broader sampling of the populations of the RP sites and more natural populations of *T. perfoliatum* and *T. aurantiacum* are needed to continue investigating the origin of the genetic diversity and population substructure, and to map out possible patterns of genetic differentiation and admixture across the landscape. Furthermore, adding *T. angustifolium, T. x eamesii*, and possibly the Asian taxa, may give further clarity to the phylogeny and genetic dynamics of the genus.

## Supporting information

S1 Fig Multiqc analysis of 38 FASTQ files of all reads per sample. (HTML)

S2 Fig Multiqc analysis of 38 BAM files with of all mapped reads per sample. (HTML)

S3 Fig Multiqc analysis of 38 FASTQ files with reads mapped at 114.404 loci used for the study. (HTML)

S4 Fig Multiqc analysis of 38 BAM files with reads mapped at 114.404 loci used for the study. (HTML)

S5 Fig Principle Component Analysis (PCA) of 26 RP samples and 5 *T. perfoliatum* and 7 *T. aurantiacum* reference samples. A) PCA of genetic diversity based on all 220,518 high quality SNPs. B) PCA of genetic diversity based on 197,029 non-singleton SNPs, after removal of singleton SNPs. A singleton SNP was defined as heterozygous genotype call in one individual and a fixed allele in all other individuals.
(PDF)

S6 Fig fastSTRUCTURE analysis of 26 RP samples and 5 *T. perfoliatum* and 7 *T. aurantiacum* reference samples. A) fastSTRUCTURE analysis of genetic diversity based on all 220,518 high quality SNPs. B) fastSTRUCTURE analysis of genetic diversity based on 197,029 non-singleton SNPs, after removal of singleton SNPs. A singleton SNP was defined as heterozygous genotype call in one individual and a fixed allele in all other individuals.
(PDF)

S7 Fig Pairwise comparisons of the genetic distance (Jaccard Inverse Distance) between individual plants within and between subpopulations, populations, and species. JID values are shown per pairwise comparison between 38 *Triosteum* individuals. JID values are calculated based on 266,257 haplotypes at 144,404 high quality loci.
(PDF)

S1 Table Statistics and QC of subsequent bioinformatics steps (read trimming, read mapping, selection of high-quality regions, and SNP filtering (genotype call missingness)). (XLSX)
