# Supplementary material for: Genome-wide molecular fingerprinting reveals local geographical genetic patterns in the North American angiosperm genus Triosteum (Caprifoliaceae)
Source: PLoS One. 2025 Jun 16;20(6):e0325657. doi: 10.1371/journal.pone.0325657 (PMC12169544; doi:10.1371/journal.pone.0325657)
Supplement: S4 Fig — (HTML) [file pone.0325657.s004.html]

MultiQC Report


# Toggle navigation v1.21

Loading report..

- General Stats
- Samtools

Toolbox

### MultiQC Toolbox

#### Apply Highlight Samples

+

Regex mode off
help
 Clear

#### Apply Rename Samples

+

Click here for bulk input.

Paste two columns of a tab-delimited table here (eg. from Excel).

First column should be the old name, second column the new name.

Add

Regex mode off
help
 Clear

#### Apply Show / Hide Samples

Hide matching samples

Show only matching samples

+

Regex mode off
help
 Clear

#### Export Plots

- Images
- Data

px

px

Aspect ratio

PNG
SVG

Plot scaling

X

Download the raw data used to create the plots in this report below:

Format:

Tab-separated
Comma-separated
JSON

Note that additional data was saved in `multiqc_data` when this report was generated.

---

##### Choose Plots

 All
 None

---


   Download Plot Images

If you use plots from MultiQC in a publication or presentation, please cite:

> **MultiQC: Summarize analysis results for multiple tools and samples in a single report**  
> *Philip Ewels, Måns Magnusson, Sverker Lundin and Max Käller*  
> Bioinformatics (2016)  
> doi: 10.1093/bioinformatics/btw354  
> PMID: 27312411

#### Save Settings

You can save the toolbox settings for this report to the browser.

 Save


---

#### Load Settings

Choose a saved report profile from the dropdown box below:

[ select ]

Load
 Delete
 Set default
 Clear default

#### Tool Citations

Please remember to cite the tools that you use in your analysis.

To help with this, you can download publication details of the tools mentioned in this report:

List of DOIs

BibTeX file

#### About MultiQC

This report was generated using MultiQC, version 1.21

You can see a YouTube video describing how to use MultiQC reports here:
https://youtu.be/qPbIlO\_KWN0

For more information about MultiQC, including other videos and
extensive documentation, please visit http://multiqc.info

You can report bugs, suggest improvements and find the source code for MultiQC on GitHub:
https://github.com/MultiQC/MultiQC

MultiQC is published in Bioinformatics:

> **MultiQC: Summarize analysis results for multiple tools and samples in a single report**  
> *Philip Ewels, Måns Magnusson, Sverker Lundin and Max Käller*  
> Bioinformatics (2016)  
> doi: 10.1093/bioinformatics/btw354  
> PMID: 27312411

---

MultiQC is developed by:

# 

A modular tool to aggregate results from bioinformatics analyses across many samples into a single report.

#### JavaScript Disabled

MultiQC reports use JavaScript for plots and toolbox functions. It looks like
you have JavaScript disabled in your web browser. Please note that many of the report
functions will not work as intended.

Loading report..

Report
generated on 2025-03-29, 23:09 CET
based on data in:
`/home/genomics/truttink/Triosteum_selected_regions_BAM`

---

×
don't show again

**Welcome!** Not sure where to start?  
Watch a tutorial video
  *(6:06)*

## General Statistics

Configure columns
 Table
Export Plot

Created with MultiQC

Copy table

 Configure columns

 Sort by highlight

 Scatter plot

 Violin plot
Export as CSV
Showing 38/38 rows and 1/3 columns.

| Sample Name | Reads | Reads mapped | % Reads mapped |
| --- | --- | --- | --- |
| RP03-01 | 23.1M | 22.9M | 99.5% |
| RP03-35 | 15.5M | 15.5M | 99.8% |
| RP03-36 | 19.9M | 19.9M | 99.8% |
| RP03-37 | 20.6M | 20.6M | 99.8% |
| RP03-38 | 32.7M | 32.6M | 99.7% |
| RP04-02 | 15.2M | 15.1M | 99.6% |
| RP05-01 | 20.0M | 19.9M | 99.6% |
| RP05-14 | 20.2M | 20.1M | 99.7% |
| RP05-15 | 16.5M | 16.5M | 99.7% |
| RP05-16 | 53.1M | 53.0M | 99.6% |
| RP05-17 | 53.8M | 53.5M | 99.6% |
| RP05-18 | 28.0M | 27.9M | 99.7% |
| RP05-30 | 41.6M | 41.4M | 99.6% |
| RP06-31 | 16.8M | 16.7M | 99.6% |
| RP06-32 | 47.6M | 47.3M | 99.5% |
| RP07-27 | 14.8M | 14.8M | 99.7% |
| RP07-28 | 25.6M | 25.5M | 99.6% |
| RP13-10 | 17.7M | 17.6M | 99.7% |
| RP13-11 | 14.1M | 14.0M | 99.6% |
| RP13-12 | 48.0M | 47.8M | 99.6% |
| RP13-13 | 64.0M | 63.7M | 99.6% |
| RP15-01 | 25.0M | 24.9M | 99.5% |
| RP15-02 | 15.2M | 15.1M | 99.8% |
| RP15-03 | 47.2M | 47.0M | 99.6% |
| RP15-04 | 49.0M | 48.8M | 99.6% |
| RP15-05 | 26.3M | 26.3M | 99.8% |
| TAUR-01 | 6.0M | 6.0M | 99.6% |
| TAUR-02 | 5.4M | 5.4M | 99.6% |
| TAUR-03 | 51.6M | 51.3M | 99.5% |
| TAUR-05 | 52.5M | 52.3M | 99.5% |
| TAUR-06 | 50.6M | 50.4M | 99.5% |
| TAUR-07 | 52.4M | 52.2M | 99.6% |
| TAUR-09 | 36.5M | 36.4M | 99.5% |
| TPER-01 | 6.3M | 6.2M | 99.6% |
| TPER-02 | 9.5M | 9.4M | 99.6% |
| TPER-03 | 48.7M | 48.5M | 99.5% |
| TPER-04 | 39.1M | 39.0M | 99.6% |
| TPER-05 | 55.0M | 54.7M | 99.5% |

×

#### General Statistics: Columns

Uncheck the tick box to hide columns. Click and drag the handle on the left to change order. Table ID: `table-general_stats_table`

Show All
Show None

| Sort | Visible | Group | Column | Description | ID | Scale |
| --- | --- | --- | --- | --- | --- | --- |
| || |  | Samtools: flagstat | Reads | Total reads in the bam file (millions) | `flagstat_total` | read\_count |
| || |  | Samtools: flagstat | Reads mapped | Reads mapped in the bam file (millions) | `mapped_passed` | read\_count |
| || |  | Samtools: flagstat | % Reads mapped | % Reads mapped in the bam file | `mapped_passed_pct` | None |

Close

## Samtools

Samtools is a suite of programs for interacting with high-throughput sequencing data.*DOI: 10.1093/bioinformatics/btp352.*

### Flagstat

This module parses the output from `samtools flagstat`. All numbers in millions.

Configure columns
 Table
Export Plot

Created with MultiQC

Copy table

 Configure columns

 Sort by highlight

 Scatter plot

 Violin plot
Export as CSV
Showing 38/38 rows and 11/11 columns.

| Sample Name | Total Reads | Total Passed QC | Mapped | Secondary Alignments | Duplicates | Paired in Sequencing | Properly Paired | Self and mate mapped | Singletons | Mate mapped to diff chr | Diff chr (mapQ >= 5) |
| --- | --- | --- | --- | --- | --- | --- | --- | --- | --- | --- | --- |
| RP03-01 | 23.1M | 23.1M | 22.9M | 0.8M | 0.0M | 22.2M | 15.1M | 21.8M | 0.4M | 6.7M | 5.1M |
| RP03-35 | 15.5M | 15.5M | 15.5M | 0.6M | 0.0M | 15.0M | 12.2M | 14.9M | 0.1M | 2.6M | 2.0M |
| RP03-36 | 19.9M | 19.9M | 19.9M | 0.7M | 0.0M | 19.2M | 15.3M | 19.1M | 0.1M | 3.8M | 2.9M |
| RP03-37 | 20.6M | 20.6M | 20.6M | 0.7M | 0.0M | 19.9M | 15.6M | 19.7M | 0.1M | 4.1M | 3.1M |
| RP03-38 | 32.7M | 32.7M | 32.6M | 1.7M | 0.0M | 31.0M | 25.1M | 30.7M | 0.3M | 5.5M | 4.3M |
| RP04-02 | 15.2M | 15.2M | 15.1M | 0.5M | 0.0M | 14.6M | 10.5M | 14.4M | 0.2M | 3.9M | 2.9M |
| RP05-01 | 20.0M | 20.0M | 19.9M | 0.7M | 0.0M | 19.3M | 13.8M | 18.9M | 0.2M | 5.1M | 3.9M |
| RP05-14 | 20.2M | 20.2M | 20.1M | 0.7M | 0.0M | 19.5M | 14.2M | 19.2M | 0.2M | 4.9M | 3.7M |
| RP05-15 | 16.5M | 16.5M | 16.5M | 0.6M | 0.0M | 15.9M | 12.2M | 15.7M | 0.1M | 3.5M | 2.7M |
| RP05-16 | 53.1M | 53.1M | 53.0M | 1.9M | 0.0M | 51.3M | 33.7M | 50.6M | 0.5M | 16.8M | 12.8M |
| RP05-17 | 53.8M | 53.8M | 53.5M | 1.9M | 0.0M | 51.8M | 32.7M | 51.0M | 0.6M | 18.2M | 13.9M |
| RP05-18 | 28.0M | 28.0M | 27.9M | 1.2M | 0.0M | 26.8M | 20.7M | 26.5M | 0.2M | 5.7M | 4.4M |
| RP05-30 | 41.6M | 41.6M | 41.4M | 1.5M | 0.0M | 40.1M | 25.0M | 39.5M | 0.5M | 14.5M | 11.0M |
| RP06-31 | 16.8M | 16.8M | 16.7M | 0.6M | 0.0M | 16.2M | 11.9M | 16.0M | 0.2M | 4.0M | 3.0M |
| RP06-32 | 47.6M | 47.6M | 47.3M | 1.6M | 0.0M | 45.9M | 28.8M | 45.1M | 0.6M | 16.3M | 12.4M |
| RP07-27 | 14.8M | 14.8M | 14.8M | 0.5M | 0.0M | 14.3M | 11.4M | 14.1M | 0.1M | 2.7M | 2.0M |
| RP07-28 | 25.6M | 25.6M | 25.5M | 0.9M | 0.0M | 24.7M | 16.0M | 24.3M | 0.3M | 8.3M | 6.3M |
| RP13-10 | 17.7M | 17.7M | 17.6M | 0.6M | 0.0M | 17.1M | 12.6M | 16.9M | 0.2M | 4.3M | 3.2M |
| RP13-11 | 14.1M | 14.1M | 14.0M | 0.5M | 0.0M | 13.6M | 9.4M | 13.4M | 0.2M | 3.9M | 2.9M |
| RP13-12 | 48.0M | 48.0M | 47.8M | 1.7M | 0.0M | 46.3M | 28.1M | 45.6M | 0.6M | 17.4M | 13.2M |
| RP13-13 | 64.0M | 64.0M | 63.7M | 2.3M | 0.0M | 61.7M | 37.2M | 60.7M | 0.7M | 23.4M | 17.7M |
| RP15-01 | 25.0M | 25.0M | 24.9M | 1.0M | 0.0M | 24.0M | 17.4M | 23.5M | 0.3M | 6.1M | 4.7M |
| RP15-02 | 15.2M | 15.2M | 15.1M | 0.5M | 0.0M | 14.6M | 11.5M | 14.5M | 0.1M | 3.0M | 2.2M |
| RP15-03 | 47.2M | 47.2M | 47.0M | 1.7M | 0.0M | 45.5M | 28.7M | 44.8M | 0.5M | 16.1M | 12.2M |
| RP15-04 | 49.0M | 49.0M | 48.8M | 1.7M | 0.0M | 47.2M | 28.4M | 46.5M | 0.5M | 18.1M | 13.6M |
| RP15-05 | 26.3M | 26.3M | 26.3M | 1.0M | 0.0M | 25.3M | 22.4M | 25.2M | 0.1M | 2.7M | 2.1M |
| TAUR-01 | 6.0M | 6.0M | 6.0M | 0.2M | 0.0M | 5.8M | 3.8M | 5.7M | 0.1M | 1.9M | 1.5M |
| TAUR-02 | 5.4M | 5.4M | 5.4M | 0.2M | 0.0M | 5.2M | 3.5M | 5.2M | 0.1M | 1.6M | 1.3M |
| TAUR-03 | 51.6M | 51.6M | 51.3M | 1.7M | 0.0M | 49.8M | 30.2M | 48.9M | 0.7M | 18.6M | 14.3M |
| TAUR-05 | 52.5M | 52.5M | 52.3M | 1.8M | 0.0M | 50.8M | 31.7M | 49.9M | 0.7M | 18.1M | 13.8M |
| TAUR-06 | 50.6M | 50.6M | 50.4M | 1.7M | 0.0M | 48.9M | 30.5M | 48.0M | 0.7M | 17.5M | 13.4M |
| TAUR-07 | 52.4M | 52.4M | 52.2M | 1.8M | 0.0M | 50.6M | 32.1M | 49.8M | 0.6M | 17.6M | 13.5M |
| TAUR-09 | 36.5M | 36.5M | 36.4M | 1.5M | 0.0M | 35.1M | 27.8M | 34.4M | 0.5M | 6.5M | 5.5M |
| TPER-01 | 6.3M | 6.3M | 6.2M | 0.2M | 0.0M | 6.0M | 3.9M | 5.9M | 0.1M | 2.0M | 1.5M |
| TPER-02 | 9.5M | 9.5M | 9.4M | 0.3M | 0.0M | 9.1M | 5.7M | 9.0M | 0.1M | 3.2M | 2.4M |
| TPER-03 | 48.7M | 48.7M | 48.5M | 3.4M | 0.0M | 45.4M | 29.4M | 44.4M | 0.7M | 14.8M | 11.4M |
| TPER-04 | 39.1M | 39.1M | 39.0M | 3.8M | 0.0M | 35.3M | 25.9M | 34.7M | 0.5M | 8.7M | 6.8M |
| TPER-05 | 55.0M | 55.0M | 54.7M | 3.7M | 0.0M | 51.3M | 35.2M | 50.3M | 0.8M | 14.8M | 11.5M |

×

#### Table-Samtools-Flagstat-Dp: Columns

Uncheck the tick box to hide columns. Click and drag the handle on the left to change order. Table ID: `table-samtools-flagstat-dp`

Show All
Show None

| Sort | Visible | Group | Column | Description | ID | Scale |
| --- | --- | --- | --- | --- | --- | --- |
| || |  |  | Total Reads | Total Reads | `flagstat_total` | read\_count |
| || |  |  | Total Passed QC | Total Passed QC | `total_passed` | read\_count |
| || |  |  | Mapped | Mapped | `mapped_passed` | read\_count |
| || |  |  | Secondary Alignments | Secondary Alignments | `secondary_passed` | read\_count |
| || |  |  | Duplicates | Duplicates | `duplicates_passed` | read\_count |
| || |  |  | Paired in Sequencing | Paired in Sequencing | `paired in sequencing_passed` | read\_count |
| || |  |  | Properly Paired | Properly Paired | `properly paired_passed` | read\_count |
| || |  |  | Self and mate mapped | Reads with itself and mate mapped | `with itself and mate mapped_passed` | read\_count |
| || |  |  | Singletons | Singletons | `singletons_passed` | read\_count |
| || |  |  | Mate mapped to diff chr | Mate mapped to different chromosome | `with mate mapped to a different chr_passed` | read\_count |
| || |  |  | Diff chr (mapQ >= 5) | Mate mapped to different chromosome (mapQ >= 5) | `with mate mapped to a different chr (mapQ >= 5)_passed` | read\_count |

Close

**MultiQC v1.21**
- Written by Phil Ewels,
available on GitHub.

This report uses HighCharts,
jQuery,
jQuery UI,
Bootstrap,
FileSaver.js and
clipboard.js.

×

### Plot Table Data

Select Column

Select Column

Please select two table columns.

Close

×

### Regex Help

Toolbox search strings can behave as regular expressions (regexes). Click a button below to see an example of it in action. Try modifying them yourself in the text box.

`^` (start of string)
`$` (end of string)
`[]` (character choice)
`\d` (shorthand for `[0-9]`)
`\w` (shorthand for `[0-9a-zA-Z_]`)
`.` (any character)
`\.` (literal full stop)
`()` `|` (group / separator)
`*` (prev char 0 or more)
`+` (prev char 1 or more)
`?` (prev char 0 or 1)
`{}` (char num times)
`{,}` (count range)

```
samp_1
samp_1_edited
samp_2
samp_2_edited
samp_3
samp_3_edited
prepended_samp_1
tmp_samp_1_edited
tmpp_samp_1_edited
tmppp_samp_1_edited
#samp_1_edited.tmp
samp_11
samp_11111
```

See regex101.com for a more heavy duty testing suite.

Close
