# Supplementary material for: Genome-wide molecular fingerprinting reveals local geographical genetic patterns in the North American angiosperm genus Triosteum (Caprifoliaceae)
Source: PLoS One. 2025 Jun 16;20(6):e0325657. doi: 10.1371/journal.pone.0325657 (PMC12169544; doi:10.1371/journal.pone.0325657)
Supplement: S5 Fig — A) PCA of genetic diversity based on all 220,518 high quality SNPs. B) PCA of genetic diversity based on 197,029 non-singleton SNPs, after removal of singleton SNPs. A singleton SNP was defined as heterozygous genotype call in one individual and a fixed allele in all other individuals. (PDF) [file pone.0325657.s005.pdf]

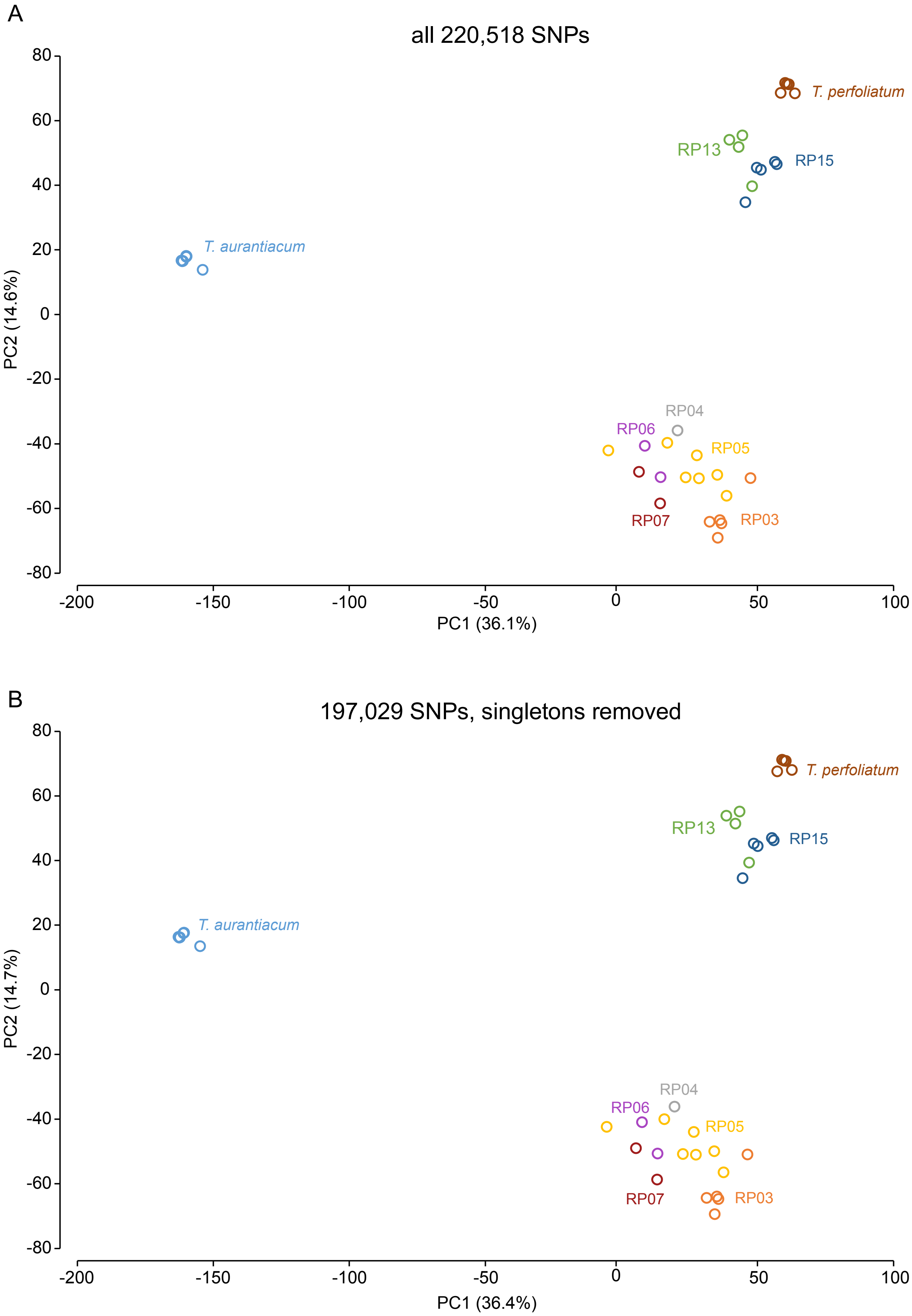

Supplemental Figure 5. Principle Component Analysis (PCA) of 26 RP samples, and five *T. perfoliatum* and seven *T. aurantiacum* reference samples. A) PCA of genetic diversity based on all 220,518 high quality SNPs. B) PCA of genetic diversity based on 197,029 non-singleton SNPs, after removal of singleton SNPs. A singleton SNP was defined as heterozygous genotype call in one individual and a fixed allele in all other individuals.
