# Supplementary figures and images for: Genome-wide molecular fingerprinting reveals local geographical genetic patterns in the North American angiosperm genus Triosteum (Caprifoliaceae)
Source: PLoS One. 2025 Jun 16;20(6):e0325657. doi: 10.1371/journal.pone.0325657 (PMC12169544; doi:10.1371/journal.pone.0325657)

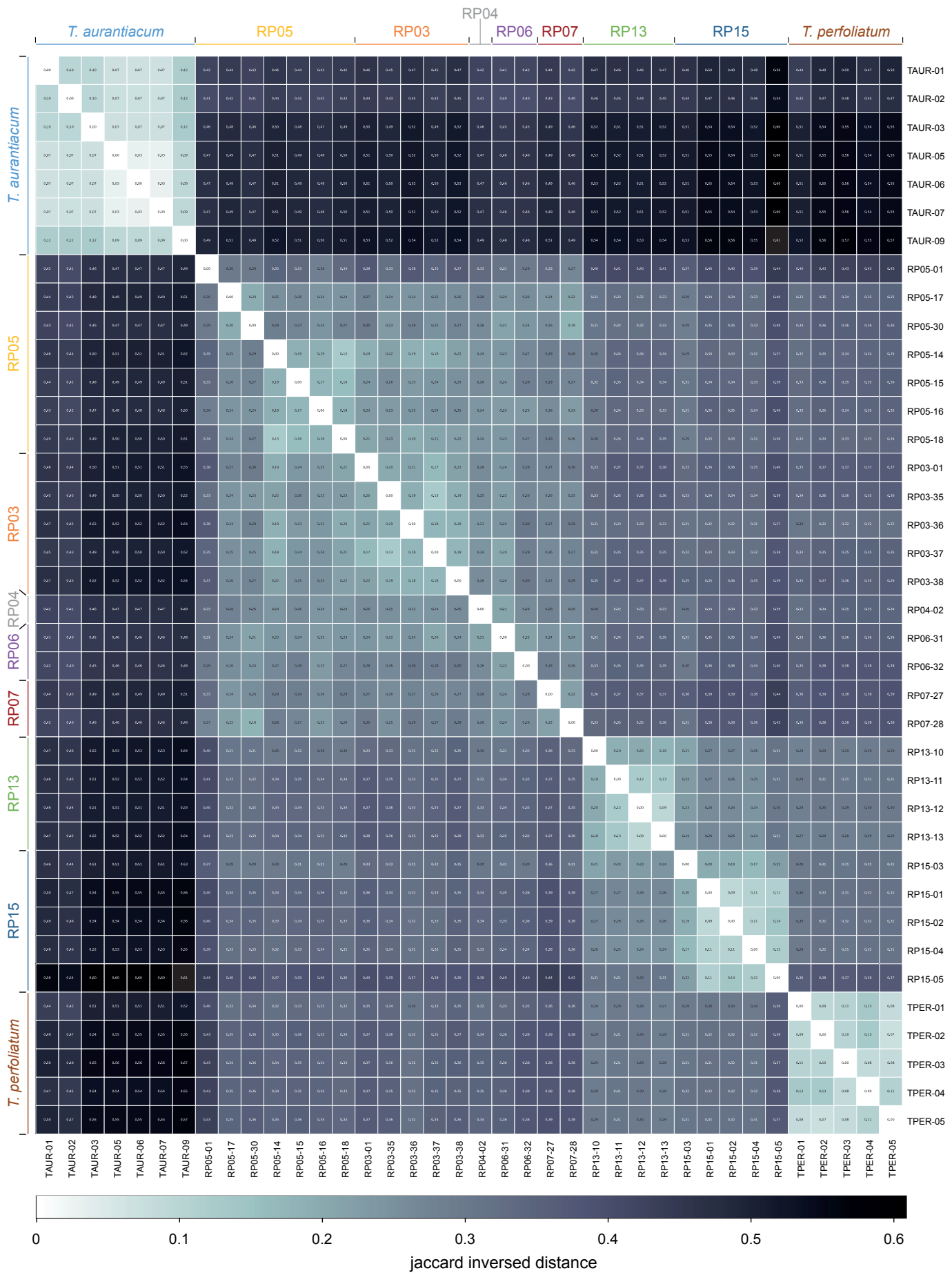

Supplement: S7 Fig — JID values are shown per pairwise comparison between 38 Triosteum individuals. JID values are calculated based on 266,257 haplotypes at 144,404 high quality loci. (PDF) [file pone.0325657.s007.pdf]
